# Near Retirement Age (≥55 Years) Self-Reported Physical Symptoms and Use of Computers/Mobile Phones at Work and at Leisure

**DOI:** 10.3390/healthcare5040071

**Published:** 2017-10-08

**Authors:** Leena Korpinen, Rauno Pääkkönen, Fabriziomaria Gobba

**Affiliations:** 1 Clinical Physiology and Neurophysiology Unit, North Karelia Central Hospital, Tikkamäentie 16, Joensuu FIN-80210, Finland; 2 TMI Rauno Pääkkönen, Timpurinkatu 7, Tampere FIN-33720, Finland; rauno.paakkonen@gmail.com; 3 Department of Public Health Sciences, University of Modena and Reggio Emilia, Via Campi 287, Modena 41125, Italy; f.gobba@unimore.it

**Keywords:** aging, physical symptoms, questionnaire, computer

## Abstract

The aim of this research is to study the symptoms and use of computers/mobile phones of individuals nearing retirement age (≥55 years). A questionnaire was sent to 15,000 Finns (aged 18–65). People who were ≥55 years of age were compared to the rest of the population. Six thousand one hundred and twenty-one persons responded to the questionnaire; 1226 of them were ≥55 years of age. Twenty-four percent of the ≥55-year-old respondents used desktop computers daily for leisure; 47.8% of them frequently experienced symptoms in the neck, and 38.5% in the shoulders. Workers aged ≥55 years had many more physical symptoms than younger people, except with respect to symptoms of the neck. Female daily occupational users of desktop computers had more physical symptoms in the neck. It is essential to take into account that, for people aged ≥55 years, the use of technology can be a sign of wellness. However, physical symptoms in the neck can be associated with the use of computers.

## 1. Introduction

In recent decades, the number of aging people in Europe has increased. By 2060, men’s mean life expectancy in the European Union is expected to be 84.5 years (an increase of 8.5 years), and women’s mean life expectancy is expected to be 89.0 years (an increase of 6.9 years). Currently, many people stop working before they reach the statutory retirement age, and the EU’s aim is to increase participation in the labor force among the 55–64 age range [1].

Musculoskeletal disorders (MSDs) are a well-known and important problem affecting the general and working population. MSDs can have a crucial impact on overall social and economic well-being [2]. In the workplace, different types of overexertion injuries can lead to work-related MSDs (WMSDs) [3]. Moreover, the increasing prevalence of obesity and rising age among workers may be associated with an increase in workplace injuries in the future [4,5,6]. Cavuoto and Nussbaum argued that considering aging alone, there are significant changes to muscle physiology that also affect muscular capacity [5]. Frontera et al. described that, in particular, older adults lose their force-generating ability, leading to strength reductions in part from slower contractile properties and loss/shrinkage of muscle fibers [7,8].

Cavuoto and Nussbaum studied the main and interactive effects of obesity and age on strength and functional performance during sustained isometric exertions involving shoulder flexion in two postures [5]. They used four groups of eight participants each: (1) non-obese young (18–25 years); (2) non-obese older (50–65 years); (3) obese young; and (4) obese older. They found that shoulder strength was ~25% higher with obesity, but there was no difference between age groups. Cavuoto and Nussbaum found, however, that obesity and age affected endurance, with the obese group having shorter endurance and the older group having longer endurance. Avin and Frey Law also reported that aging has been associated with longer endurance times and increased fatigue resistance in static tasks [9]. On the contrary, Mehta and Cavuoto summarized that, to date, none of these studies have systematically considered individual differences based on the level of both obesity and age across a range of workloads [10].

Cheng et al. studied the age-specific patterns of self-rated health (SRH) and burnout and their correlations with self-reported disease symptoms [11]. Their goal was to investigate the moderating effects of age on the relationships between psychosocial work conditions and these two health measures. Their study included 20,454 male and 16,875 female employees from two representative surveys conducted in 2007 and 2010 in Taiwan. They found that older workers were at higher risk for poor SRH, correlating with the presence of multiple disease symptoms. They concluded that SRH and burnout were differentially related to age, and found evidence that age moderates the relationship between psychosocial work conditions and health.

Computer use is thought to be associated with musculoskeletal symptoms, e.g., among office workers. Risk is higher among workers who experience high work strain, continuous keyboard or mouse use, high muscle tension, and previous MSDs [12,13,14,15,16,17].

A further problem related to WMSDs is the recent technological developments that have introduced new technological equipment into the workplace, resulting in new types of human–computer interaction. In light of this new challenge, we performed a study on the possible influences of new technological equipment on the health of the working-age population. We sent a questionnaire to Finns, which was divided into six sections: (1) background information, such as age, gender, marital status, education, occupation, and home county; (2) familiarity with and use of given technological devices at leisure and at work; (3) physical loading and ergonomics; (4) psychological welfare; (5) accidents and close-call situations; and (6) an open-ended question: “other observations concerning technology and health”. The details of the questionnaire and the results of the respondents’ self-reported physical and mental symptoms have already been published [18,19].

The use of computers/mobile phones can cause different problems or increase symptoms among members of the working population who are close to retirement age (≥55 years). The goals of this study were to determine the correlation between self-reported physical symptoms (aches, pain, or numbness) and computer/mobile phone use in the near retirement age (≥55 years) working population, and to analyze whether these symptoms were specifically associated with the use of desktop computers and mobile phones. We hypothesized that these new devices may increase the risk of developing physical symptoms related to, e.g., poor working postures in the near retirement age (≥55 years) working population. In previous studies, we considered the relationship between self-reported mental symptoms and background information [18,19].

## 2. Methods

In October 2002, a questionnaire was sent to 15,000 Finns. As the study focused on the working age population, only people aged 18–65 were included. Names and addresses were obtained at random from the Finnish Population Registration Centre. The study design was approved by an Ethics Committee (Pirkanmaa Health District, Finland, decision R02099).

For the statistical analyses, we included only those who were 55 years of age or older (≥55) and focused on the following questions regarding physical and mental symptoms: Question (13), “Have you had an ache, pain, or numbness in the following body part during the last 12 months? (a) wrists and fingers; (b) elbows and forearms; (c) neck; (d) shoulders; (e) hip and lower back; (f) feet”; and Question (16), “Have you suffered from (a) sleep disorders/disturbances; (b) depression; (c) exhaustion at work; (d) substance addiction; (e) anxiety; or (f) fear situations during the last 12 months?” The options for Questions 13 and 16 were “cannot say”, “not at all”, “sometimes”, “quite often”, “often”, and “very often”.

In the first analysis of Questions 13 and 16, we used the Mann-Whitney U-Test to compare independent samples. We compared those who were ≥55 years old to younger workers (<55 years), dividing them into following groups: (1) all respondents; (2) all workers; (3) daily desktop computer (PC) users for leisure; (4) daily desktop computer users at work; (5) female workers who use a desktop computer daily at work; (6) male workers who use a desktop computer daily at work; (7) entrepreneurs; (8) upper-level white-collar workers; (9) lower-level white-collar workers; and (10) blue-collar workers.

In the second analysis of Questions 13 and 16, we used the Mann-Whitney U-Test to compare independent samples of those who were ≥55 years old and used a desktop computer daily for leisure with those who were ≥55 years old and did not use a computer for leisure. We also examined the samples in terms of computer use for work, laptop use, and mobile phone use. Likewise, we also analyzed the differences between men and women who were ≥55 years old in terms of daily desktop computer use and mobile phone use at work. A *p*-value of 0.05 was chosen for this study. All statistical analyses were done using IBM SPSS Statistics versions 22 and 23.

## 3. Results

Six thousand one hundred twenty-one working age individuals responded to the questionnaire (a response rate of 41%). The data included 1226 people who were 55 years of age or older. In this age group, there were 656 women and 568 men, and Table 1 presents their background information and the number of “quite often”, “often”, and “very often” responses with regard to the symptoms. Among people in the group aged ≥55 years, 24.0% of the respondents used a desktop computer daily for leisure (women: 21.0%; men: 27.3%).

With respect to Question (13), “Have you had an ache, pain, or numbness in the following body part during the last 12 months? (a) wrists and fingers; (b) elbows and forearms; (c) neck; (d) shoulders; (e) hip and lower back; (f) feet?”, 47.8% of respondents in the ≥ 55 group experienced symptoms quite often or more in their neck, 38.5% experienced symptoms in their shoulders, 38.0% in their hip and lower back, and 37.3% in their feet. In this group, 21.1% of respondents reported experiencing sleep disorders/disturbances quite often or even more frequently.

Table 2 shows the results of the Mann-Whitney U-Test for Questions 13 and 16, comparing those who were ≥55 years of age and others (<55 years) according to the following groups: (1) all respondents; (2) all workers; (3) daily desktop computer (PC) users for leisure; (4) daily desktop computer users at work; (5) female workers who use a desktop computer daily at work; (6) male workers who use a desktop computer daily at work; (7) entrepreneurs; (8) upper-level white-collar workers; (9) lower-level white-collar workers; and (10) blue-collar workers. The idea was to study the differences between ≥ 55-year-olds and younger individuals (<55 years).

In group 1 (all respondents), there were significant differences between all symptoms, except for fear situations. The ≥55-year-old people reported experiencing all other physical symptoms more frequently than the younger group, except for symptoms in the neck. The <55 group self-reported more depression, exhaustion at work, substance addiction, and anxiety than the ≥55 group. In group 2 (all workers), there were significant differences between physical symptoms, except for symptoms in the hip and lower back. There was also a significant difference in terms of responses to Question 16a (sleep disorders/disturbances). The ≥55-year-old workers had more physical symptoms in their wrists and fingers, elbows and forearms, shoulders, and feet, but the <55-year-old workers had more symptoms in their neck. The ≥55-year-old workers also had more sleep disorders/disturbances than the younger workers. In group 3 (daily desktop computer (PC) users for leisure), the <55-year-old respondents reported feeling more depression and exhaustion at work. In group 4 (daily desktop computer users at work) and group 5 (female workers who use a desktop computer daily at work), the ≥55-year-old workers reported more physical symptoms affecting their elbows and forearms, and feet.

In group 6 (male workers who use a desktop computer daily at work) and group 8 (upper-level white-collar workers), the ≥55-year-old workers had more physical symptoms in their feet. In group 9 (lower-level white-collar workers), the ≥55-year-old workers experienced more physical symptoms in their elbows and forearms, and feet. In group 10 (blue-collar workers), the ≥55-year-old workers had more physical symptoms in their wrists and fingers, elbows and forearms, shoulders, and feet. The ≥ 55-year-old blue-collar workers reported more sleep disorders/disturbances and exhaustion at work than the younger blue-collar workers.

Table 3 shows the results related to the ≥55-year-old respondents, using the Mann-Whitney U-Test for Question 13 to compare: (1) those who used a desktop computer daily for leisure and those who did not; (2) those who used a laptop daily and those who did not; and (3) those who used a mobile phone daily and those who did not.

Table 3 also includes the same comparisons in terms of women, men, and workers, and comparisons 1 and 3 in terms of female and male workers. Table 4 shows similar comparisons of the ≥55-year-old respondents using the Mann-Whitney U-Test for Question 16.

Considering the data for the ≥55-year-old respondents, those who used a desktop computer daily for leisure reported fewer physical symptoms in their wrists and fingers, elbows and forearms, shoulders, hip and lower back, and feet than other users. The results were similar for ≥55-year-old laptop users. Daily users of laptops had fewer physical symptoms in their wrists and fingers, elbows and forearms, neck, shoulders, hip and lower back, and feet than more casual users or non-users. Daily users of mobile phones and female daily users of desktop computers had fewer symptoms in their feet than others. Male daily users of desktop computers had fewer physical symptoms in their wrists and fingers, elbows and forearms, shoulders, hip and lower back, and feet than other men. Male daily users of laptops had fewer physical symptoms in their elbows and forearms, shoulders, hip and lower back, and feet than other men. The male daily users of mobile phones had fewer physical symptoms in their shoulders and feet than other men.

In the group consisting of workers, daily users of desktop computers had fewer physical symptoms in their shoulders, hip and lower back, and feet than others. Daily occupational users of laptops had fewer physical symptoms in their wrists and fingers, elbows and forearms, neck, shoulders, and feet than others. Daily occupational users of mobile phones had fewer physical symptoms in their elbows and forearms, neck, and shoulders than others. Female daily occupational users of desktop computers had more physical symptoms in their neck, but fewer in their feet than others. Male daily occupational users of desktop computers had fewer physical symptoms in their wrists and fingers, elbows and forearms, shoulders, hip and lower back, and feet than others.

Table 4 shows that all (≥55 years) daily users and female users of desktop computers or mobile phones were more exhausted at work than others (≥55 years). In addition, all (≥55 years) daily users and female users of laptops self-reported more substance addiction than others (≥55 years). Occupational daily users (≥55 years) of mobile phones self-reported fewer sleep disorders/disturbances, but more substance addiction than others (≥55 years). Occupational daily users (≥55 years) of laptops self-reported more substance addiction than others (≥55 years). Female daily occupational users of desktop computers had more anxiety than others, and female daily occupational users of mobile phones self-reported more substance addiction than others. Male daily occupational users of mobile phones had fewer sleep disorders/disturbances and less depression and exhaustion at work than others.

## 4. Discussion

The population was 15,000 Finns, and the number of responses was 6121—a large enough number to conduct analyses on the subgroups. For these analyses, 1226 participants who were 55 years of age or older, were used. However, the approach applied in this study has some limitations. The questionnaire and questions could have influenced participants, and only those who were active might have sent back the questionnaire. Furthermore, opinions can change quite quickly as technology develops. Not all participants might have understood their symptoms in the same way, and the questionnaire did not include all possible questions or symptoms. In addition, some of the older respondents were already retired but based their answers on the time period when they had been working. This may have made the questionnaire more difficult for them to fill out. It is also important to note that the use of computers (especially laptops, other portable computers, and mobile phones) has increased from the time our data was collected (2002–2003), and new devices, such as tablets, e-readers, and smartphones, have been more recently introduced. These limitations must be considered when comparing our results with more recent results in the literature.

Table 2 shows some interesting results with respect to the respondents’ physical symptoms. The people and groups of workers ≥ 55 years of age experienced almost all physical symptoms significantly more often, except for symptoms in the neck. Younger respondents had more neck symptoms. This finding is difficult to explain. One possible explanation is that older workers have found solutions to prevent physical symptoms in their neck. Another possibility is that other symptoms require older individuals to take more breaks at work. In a previous article, we reported the symptoms of all participants [19]. In total, 19.9% of respondents reported having pain, aches, or numbness quite often (or more frequently) in their wrists or fingers, 13.5% reported having physical symptoms in their elbows or forearms, 49.8% in their neck, 30.2% in their shoulders, 33.0% in their lower back, and 24.8% in their feet [19].

Another interesting finding was that those who were younger than 55 years reported experiencing more depression, exhaustion at work, substance addiction, and anxiety than those who were ≥55 years. However, the ≥55-year-old workers had more sleep disorders/disturbances than younger workers. It is possible that the older respondents characterized their mental symptoms differently from the younger respondents. The details of the mental symptoms of all respondents have also been reported in an earlier article [18].

Table 3 also shows an interesting finding. The ≥ 55-year-old respondents who used desktop computers, laptops, or mobile phones daily for leisure or at work had fewer physical symptoms. One possible explanation is that these older persons are more active or have a greater sense of well-being, and thus are able to use different technology more easily than others who are less active. In this case, the technology perhaps supports their well-being. However, the group of female daily occupational users of desktop computers reported more physical symptoms in their neck. It would seem that in this particular group of users, computer use can increase the incidence of neck symptoms. Table 4 also shows that this group experiences more exhaustion at work than others. These symptoms and the differences between them require further study, especially when trying to find technological solutions for the better well-being of the aging population. This is a very important issue, particularly in light of the discussions to raise the retirement age.

Examining these results, we can only partly accept our hypothesis that these new devices may increase the risk of developing physical symptoms related to, e.g., poor working postures in the near retirement age (≥55 years) working population. The female daily occupational users of desktop computers had more physical symptoms in their neck, but had no significant differences or even fewer physical symptoms with respect to the other symptoms.

The focus of this paper is primarily on the well-being and health of the near retirement age (≥ 55 years) working population. There are certainly other issues that influence a person’s well-being when he or she is close to retirement age, e.g., the nature of the work, the resiliency of the person, the person’s family situation, the company’s economic situation, and the structure of the pension.

## 5. Conclusions

The results of this study indicated that 47.8% of Finnish working population aged ≥ 55 years experienced pain, numbness, and aches in their neck quite often or even more frequently. Furthermore, they frequently had other symptoms: 38.5% had symptoms in the shoulders; 38.0% suffered symptoms in the hip and lower back; and 37.3% had symptoms in the feet. In addition, 21.1% of them reported having sleep disorders/disturbances quite often or even more frequently. The ≥ 55-year-old population (all respondents) and ≥ 55-year-old workers had more physical symptoms than younger respondents, except for symptoms in the neck. However, the ≥ 55-year-old individuals who used desktop computers, laptops, or mobile phones daily for leisure or at work had fewer physical symptoms than the ≥ 55-year-old people who did not use such devices daily. Only female daily occupational users of desktop computers had more physical symptoms in the neck and greater self-reported exhaustion at work. In the future, it is essential to take into account that, to those ≥ 55 years old, the active use of new technology can be a sign of wellness.

## Figures and Tables

**Table 1:** Summary of background information and the results of all ≥55-year-old participants. The daily use of different devices/computers at leisure, physical and mental symptoms, (number of positive answers including answers “quite often”, “often”, and “very often”).

Question Topics and Choices	Women (%) (n = 656)	Men (%) (n = 568)	Total (%) (n = 1226)
**Marital Status**			
single (n = 84)	7.3	6.3	6.9
married or live-in (n = 951)	72.8	83.5	77.7
divorced (n = 113)	10.6	7.7	9.2
widow or widower (n = 75)	9.2	2.5	6.1
**Education**			
comprehensive school (n = 415)	33.9	34.2	34.1
matriculation (n = 36)	3.2	2.7	3.0
vocational school (n = 312)	25.0	26.4	25.6
vocational high school (n = 285)	25.0	21.6	23.4
university (n = 170)	12.7	15.2	14.0
**Occupation**			
none ^a^ (n = 11)	0.8	1.1	0.9
enterpriser (n = 121)	6.1	14.3	9.9
farmer (n = 79)	5.5	7.6	6.5
upper-level white-collar workers ^b^ (n = 240)	16.3	23.5	19.7
lower-level white-collar workers ^c^ (n = 329)	33.2	20.0	27.0
blue-collar workers ^d^ (n = 359)	31.3	27.2	29.5
home work, student (n = 11)	1.5	0.2	0.9
other (n = 69)	5.2	6.2	5.7
**Use at Leisure (Daily)**			
mobile phone (n = 787)	61.3	69.1	64.9
desktop computer (n = 284)	21.0	27.3	24.0
internet (n = 152)	9.8	16.1	12.8
portable computer (n = 39)	1.0	5.8	3.3
**Experienced Pain, Numbness, or Aches ^e^**			
in wrists and fingers (n = 334)	30.8	25.4	28.3
in elbows or forearms (n = 241)	24.3	16.7	20.9
in neck (n = 573)	53.7	40.9	47.8
in shoulders (n = 454)	40.5	36.2	38.5
in hip and lower back (n = 449)	40.5	35.1	38.0
in feet (n = 445)	40.9	33.0	37.3
**Mental Symptoms ^e^**			
sleeping disorders/disturbances (n = 253)	22.9	19.1	21.1
depression (n = 98)	9.4	7.1	8.3
exhaustion at work (n = 183)	15.3	15.8	15.5
substance addiction (n = 21)	1.1	2.5	1.7
anxiety (n = 64)	6.5	4.2	5.4
fear situations (n = 33)	3.1	2.5	2.8

^a^ never had an occupation; ^b^ administrative or managerial duties, designing, research, teaching; ^c^ clerical duties and supervision; ^d^ industrial workers, distributive and service trade; ^e^ self-reported symptoms quite often or more.

**Table 2:** The results of Mann-Whitney U-Test for Questions 13 and 16 between the ≥ 55-year-old persons and others (< 55 years) using the following groups: (1) all respondents; (2) all workers; (3) daily desktop computer (PC) users at leisure; (4) workers who use a desktop computer daily at work; (5) female workers who use a desktop computer daily at work; (6) male workers who use a desktop computer daily at work; (7) entrepreneurs; (8) upper-level white-collar workers; (9) lower-level white-collar workers; and (10) blue-collar workers.

**Q13: Have You Had an Ache, Pain, or Numbness in the Following Body Part during the Past 12 Months?**						
Participants	Wrists and Fingers	Elbows and Forearms	Neck	Shoulders	Hip and Lower Back	Feet
All respondents	<0.001 *	<0.001 *	0.001 *	<0.001 *	0.013 *	<0.001 *
All workers	0.001 *	<0.001 *	0.032 *	0.001 *	n.s.	<0.001 *
Daily PC users at leisure	n.s.	n.s.	n.s.	n.s.	n.s.	n.s.
Workers; daily PC users ^1^	n.s.	0.001 *	n.s.	n.s.	n.s.	0.004 *
Female workers; PC users ^1^	n.s.	<0.001 *	n.s.	n.s.	n.s.	0.029 *
Male workers; PC users ^1^	n.s.	n.s.	n.s.	n.s.	n.s.	0.025 *
Entrepreneurs	n.s.	n.s.	n.s.	n.s.	n.s.	n.s.
Upper-level white-collar workers	n.s.	n.s.	n.s.	n.s.	n.s.	0.035 *
Lower-level white-collar workers	n.s.	0.008 *	n.s.	n.s.	n.s.	0.001 *
Blue-collar workers	<0.001 *	<0.001 *	n.s.	< 0.001 *	n.s.	<0.001 *
**Q16: Have You Suffered from One of the Following Symptoms in the Past 12 Months?**						
Participants	Sleeping Disorders/Disturbances	Depression	Exhaustion at Work	Substance Addiction	Anxiety	Fear Situations
All respondents	0.011 *	<0.001 *	<0.001 *	0.003 *	<0.001 *	n.s.
All workers	0.002 *	n.s.	n.s.	n.s.	n.s.	n.s.
Daily PC users at leisure	n.s.	0.027 *	0.009 *	n.s.	n.s.	n.s.
Workers, daily PC users ^1^	n.s.	n.s.	n.s.	n.s.	n.s.	n.s.
Female workers, PC users ^1^	n.s.	n.s.	n.s.	n.s.	n.s.	n.s.
Male workers, PC users ^1^	n.s.	n.s.	n.s.	n.s.	n.s.	n.s.
Entrepreneurs	n.s.	n.s.	n.s.	n.s.	n.s.	n.s.
Upper-level white-collar workers	n.s.	n.s.	n.s.	n.s.	n.s.	n.s.
Lower-level white-collar workers	n.s.	n.s.	n.s.	n.s.	n.s.	n.s.
Blue-collar workers	0.001 *	n.s.	0.020 *	n.s.	n.s.	n.s.

Notes. * *p* < 0.05, asymptotic significance; ^1^ workers who use a desktop computer daily at work, n.s.: not significant.

**Table 3:** The results of the ≥55-year-old persons using Mann-Whitney U-Test for Question 13. The comparisons are the following: (1) persons who used a desktop computer daily at leisure and non-users, (2) daily users of laptops and non-users, (3) daily users of a mobile phone and non-users. This table also includes the same comparisons for women and men and workers, and comparisons 1 and 3 for female workers and male workers.

Have You Had an Ache, Pain, or Numbness in the Following Body Part during the Past 12 Months?						
Participants	Wrists and Fingers	Elbows and Forearms	Neck	Shoulders	Hip and Lower Back	Feet
**All ≥55 years (n = 1226)**						
PC Users ^1^—non-users	0008 *	0.001 *	n.s.	0.003 *	<0.001 *	<0.001 *
Laptop users ^2^—non-users	0.006 *	<0.001 *	0.008 *	0.004 *	0.009 *	<0.001 *
MP users ^3^—non-users	n.s.	n.s.	n.s.	0.021 *	n.s.	0.005 *
**Women; ≥55 years (n = 656)**						
PC Users ^1^—non-users	n.s.	n.s.	n.s.	n.s.	n.s.	<0.0.01 *
Laptop users ^2^—non-users	n.s.	n.s.	n.s.	n.s.	n.s.	n.s.
MP users ^3^—non-users	n.s.	n.s.	n.s.	n.s.	n.s.	n.s
**Men; ≥55 years (n = 568)**						
PC Users ^1^—non-users	0.006 *	<0.001	n.s.	0.001 *	<0.001 *	<0.001 *
Laptop users ^2^—non-users	n.s.	0.001 *	n.s.	0.045 *	0025 *	0.005 *
MP users ^3^—non-users	n.s.	n.s.	n.s.	0.044 *	n.s.	0.023 *
**Workers; ≥55 years (n = 662)**						
PC Users ^4^—non-users	n.s.	n.s.	n.s.	0.046 *	0.019 *	<0.001 *
Laptop users ^5^—non-users	0.007 *	0.001 *	0.003 *	0.006 *	n.s.	0.014 *
MP users ^6^—non-users	n.s.	0.007 *	0.003 *	0.011 *	n.s.	n.s.
**Female workers; ≥55 years (n = 356)**						
PC Users ^4^—non-users	n.s.	n.s.	0.035 *	n.s.	n.s.	0.006 *
MP users ^6^—non-users	n.s.	n.s.	n.s.	n.s.	n.s.	n.s.
**Male workers; ≥55 years (n = 304)**						
PC Users ^4^—non-users	0.036 *	<0.001 *	n.s.	<0.001 *	0.004 *	0.003 *
MP users ^6^—non-users	n.s.	0.029 *	n.s.	n.s.	n.s.	n.s.

* = significant at *p* < 0.05; PC users 1 = persons who use a desktop computer daily at leisure; laptop users 2 = persons who use a portable computer or a mini-computer daily at leisure; MP users 3 = persons who use a mobile phone daily at leisure, PC users 4 = workers who use a desktop computer daily at work; laptop users 5 = worker who use a portable computer or a mini-computer daily at work; MP users 6 = workers who use a mobile phone daily at work.

**Table 4:** The results of the ≥55-year-old persons using Mann-Whitney U-Test for Question 16. The comparisons are the following: (1) persons who used a desktop computer daily at leisure and non-users, (2) daily users of laptops and non-users, (3) daily users of the mobile phone and non-users. This table also includes the same comparisons for women and men and workers, and comparison 1 and 3 for female workers and male workers.

Have You Suffered from One of the Following Symptoms in the Past 12 Months?						
Participants	Sleeping Disorders/Disturbances	Depression	Exhaustion at Work	Substance Addiction	Anxiety	Fear Situations
**All ≥ 55 Years (n = 1226)**						
PC Users ^1^—non-users	n.s	n.s	0.008 *	n.s	n.s	n.s
Laptop users ^2^—non-users	n.s	n.s	n.s	0.002 *	n.s	n.s
MP users ^3^—non-users	n.s	n.s	0.007 *	n.s	n.s	n.s
**Women; ≥ 55 Years (n = 656)**						
PC Users ^1^—non-users	n.s	n.s	0.008 *	n.s	n.s	n.s
Laptop users ^2^—non-users	n.s	n.s	n.s	<0.001 *	n.s	n.s
MP users ^3^—non-users	n.s	n.s	0.009 *	n.s	n.s	n.s
**Men; ≥ 55 Years (n = 568)**						
PC Users ^1^—non-users	n.s	n.s.	n.s.	n.s.	n.s.	n.s.
Laptop users ^2^—non-users	n.s.	n.s.	n.s.	n.s.	n.s.	n.s.
MP users ^3^—non-users	n.s.	n.s.	n.s.	n.s.	n.s.	n.s.
**Workers; ≥ 55 Years (n = 662)**						
PC Users ^4^—non-users	n.s.	n.s.	n.s.	n.s.	n.s.	n.s.
Laptop users ^5^—non-users	n.s.	n.s.	n.s.	0.018 *.	n.s.	n.s.
MP users ^6^—non-users	0.031 *	n.s.	n.s.	0.020 *	n.s.	n.s.
**Female Workers; ≥ 55 Years (n = 356)**						
PC Users ^4^—non-users	n.s.	n.s.	n.s.	n.s.	0.040 *	n.s.
MP users ^6^—non-users	n.s.	n.s.	n.s.	0.024 *	n.s.	n.s.
**Male Workers; ≥ 55 Years (n = 304)**						
PC Users ^4^—non-users	n.s.	n.s.	n.s.	n.s.	n.s.	n.s.
MP users ^6^—non-users	0.007 *	0.012 *	0.040 *	n.s.	n.s.	n.s.

* = significant at *p* < 0.05; PC users 1 = persons who use a desktop computer daily at leisure; laptop users 2 = persons who use a portable computer or a mini-computer daily at leisure; MP users 3 = persons who use a mobile phone daily at leisure; PC users 4 = workers who use a desktop computer daily at work; laptop users 5 = workers who use a portable computer or a mini-computer daily at work; MP users 6 = workers who use a mobile phone daily at work.
